# Current situations of animal data recording, dairy improvement infrastructure, human capacity and strategic issues affecting dairy production in sub-Saharan Africa

**DOI:** 10.1007/s11250-019-01871-9

**Published:** 2019-04-03

**Authors:** O. Opoola, R. Mrode, G. Banos, J. Ojango, C. Banga, G. Simm, M. G. G. Chagunda

**Affiliations:** 1 The Roslin Institute and The Royal (Dick) School of Veterinary Studies, University of Edinburgh, Easter Bush, Midlothian, UK; 2 Scotland’s Rural College (SRUC), Edinburgh, UK; 3 International Livestock Research Institute (ILRI), Nairobi, Kenya; 4 Agricultural Research Council (ARC), Pretoria, South Africa; 5 Animal Breeding and Husbandry in the Tropics and Subtropics, University of Hohenheim, Stuttgart, Germany

**Keywords:** Across-country collaboration, Dairy farming systems, Genetic gain, Joint genetic assessments, Milk yield, Sub-Saharan Africa

## Abstract

An online survey on the state of existing dairy data, dairy improvement infrastructure and human capacity in sub-Saharan Africa (SSA) was undertaken with the aim of assessing whether the state of existing animal recording, dairy improvement methods and key issues facing dairy production together with means of addressing the issues differ among countries and regions of SSA. Respondents comprised experts and practitioners in livestock production and genetic resources from research institutes, animal breeding companies, universities, non-governmental organisations and government agricultural ministries. The main dairy farming system in which the respondents were involved was mixed crop-livestock system (30.2%), and this was mainly practised in the private land tenure system (46.3%). Data were analysed using linear model and paired Student *t* test in R software package. Respondents identified key issues affecting dairy production as poor genetic assessment of imported exotic breeds and crosses in Africa (62.3%), fluctuations in milk prices within both the formal and informal markets (50.9%), no comprehensive sire ranking systems (39.6%), housing and health management regimes which adversely affect milk yield (32.1%), poor market networks for dairy products (25.5%), poor feeding (13.3%), inadequate genetic technologies (9.4%) and poor animal performance recording systems (9.4%). Respondents emphasised the need for updated breeding policies, sire ranking systems, adequate farm management systems, capacity building, across-country collaborations and joint genetic assessments of dairy breeds found in sub-Saharan Africa. The current situation of dairy production though similar for the different countries, differed in order of emphasis and magnitude across the countries and regions in sub-Saharan Africa.

## Introduction

Smallholder farms, those with approximately 2 ha of land per farm, contribute up to 90% of the agricultural produce (FAO 2015; Martin-Collado et al. 2015). There are 59 million dairy cattle in SSA (FAOSTAT 2015). Cows’ milk accounts for 80% of total milk (FAOSTAT 2016), of which half is produced in Eastern Africa, followed by Central and Southern Africa while Western Africa produces the least amount of milk. The global demand for animal-derived products is increasing by more than 2% (Yáñez-Ruiz and Martín-García 2016) and by more than 3% in SSA per annum (FAOSTAT 2016). Holstein-Friesian is the main exotic dairy breed used for milk production in SSA. However, other breeds such as Jersey, Guernsey, Brown Swiss, Fleckvieh and Ayrshire are also found.

In many developed countries, there has been tremendous improvement in desirable quantitative traits since the implementation of genetic selection principles to livestock production. This has led to increased availability of milk, meat, eggs and other animal products in these countries. Over time, the rate of genetic improvement has been stable in the developed countries with fewer changes that could be sustainable for future livestock and the growing population. However, genetic improvement has not been widely implemented in SSA. This is largely due to lack of well-defined breeding goals and strategies, limited pedigree and performance data recording, lack of human capacity and inadequate dairy management practises (Missanjo 2010). Recent initiatives and improvement methods in SSA may provide opportunities for efficient animal data recording and implementation of organised breeding schemes. Also, with recent advances in statistical genetics, there is an opportunity to develop new approaches to livestock improvement, potentially suitable for application in SSA.

Despite the rapid influx of foreign (exotic) high-producing breeds into Africa, the dairy sector is still faced with huge productivity gaps (Ojango et al. 2017). In order to contribute towards bridging these productivity gaps, there is a need for a better understanding of the situation not only from the viewpoint of farmers but also the experts that work in the livestock sector. Therefore, the objectives of this study were to (i) determine the current status of dairy production systems, infrastructure and genetic improvement methods in different countries and regions of SSA and (ii) assess strategic issues affecting the dairy sector in different countries and regions of SSA and outline possible solutions to mitigating the issues.

## Materials and methods

A survey was carried out using an online questionnaire. Questionnaire recipients were participants in different scientific conferences and workshops related to dairying and genetics that had taken place in Africa over a 16-year period (2000–2016). The conferences and workshops were randomly selected. All names on the delegate lists were contacted as respondents. A total of 496 recipients from the following 15 countries were contacted: Burkina Faso (BF), Burundi (BR), Cameroon (CA), Ethiopia (ET), Gambia (GA), Ivory Coast (IC), Kenya (KE), Malawi (ML), Nigeria (NG), Senegal (SE), South Africa (SA), Sudan (SU), Tanzania (TZ), Uganda (UG) and Zimbabwe (ZW). The delegates were predominantly trained scientists and professionals working with farmers. They described themselves in the following categories: animal scientists, livestock extension workers, animal nutritionists, geneticists, animal husbandry workers, researchers and animal breeders. The questionnaire was implemented using Snap WebHost® software and sent to the recipients via e-mail. The e-survey was active for a period of 90 days after which no further responses were accepted. A reminder e-mail was sent automatically every 28 days after first receipt. The main themes in the survey included: dairy policies, dairy production challenges, current levels of production and fertility, breeding strategies, breeds and genotypes (crossbreeds) in use, data recording systems, livestock improvement infrastructure, human capacity and genetic evaluation methods. The survey had 22 questions which were a combination of open-ended, close-ended, structured and unstructured questions.

### Statistical analysis

Survey data were analysed by country and region with the latter defined as Eastern Africa, Southern Africa and Western Africa. The survey data were analysed using descriptive statistics that included means, percentages and frequencies of which contingency tables and crosstabs of variables were generated. A one-way analysis of variance was used to test differences between variables/groups in the respondent’s countries and the three regions of Africa. Hypotheses tested were:

- Null hypothesis (*H*_0_) = the status of animal recording, dairy improvement methods, dairy production issues and methods used to alleviate challenges in dairy production were the same across countries and regions of Africa.
- Alternative hypothesis (*H*_A_) = the status of animal recording, dairy improvement methods, dairy production issues and methods used to alleviate challenges in dairy production were not the same across countries and regions of Africa.

The following is the linear model that was implemented to test the hypotheses:

1 $$ Y= X\beta +e, $$

where

*Y*: dependent variable (current dairy status variable)
*Xβ*: independent variable (region or country as fixed effect 3 or 15 levels, respectively)
*e*: error term

Marginal means and standard errors were estimated for each of the current dairy status in the three regions specified. The R software package (R Core Team 2013) was used in the analysis. Student *t* test was done to determine pairwise comparison of across-country existing dairy status where the model is given as:

2 $$ t=\frac{M_1-{M}_2}{\sqrt{{\left({SE}_1\right)}^2+{\left({SE}_2\right)}^2}}, $$

where

*t*: test statistic for differences between group means
*M*_1_: group means for existing dairy status in region 1/ country 1
*M*_2_: group means for existing dairy status in region 2/ country 2
*SE*_1_: standard error for group means for existing dairy status in region 1/ country 1
*SE*_2_: standard error for group means for existing dairy status in region 2/ country 2

## Result

Out of the 496 questionnaire that were sent, a total of 70 responses were obtained. These were those that voluntarily responded to the survey and hence represent a random, self-selected and representative sample. The respondents were from 15 countries namely, Burundi (BR), Burkina Faso (BF), Ivory Coast (IC), Cameroon (CA), Ethiopia (ET), Kenya (KE), Gambia (GA), Malawi (ML), Nigeria (NG), Senegal (SE), Sudan (SU), South Africa (SA), Tanzania (TZ), Uganda (UG) and Zimbabwe (ZW). Most respondents were from KE, NG and TZ comprising more males (90%) than females (10%) involved in various occupations. They were associated with different farming systems and land tenure systems in which dairy production is practised. BR, BF, GA, SU and SE had the lowest numbers of respondents. Although the study did not access all stakeholders involved with dairy production in SSA, the data were obtained from animal experts and scientists who work directly with different stakeholders including farmers, producers, processors, distributers, breeding input and services suppliers, development and extension agents and policy makers. The general status of dairy production across the countries, as given by the respondents of the survey, is summarised in Table 1.

**Table 1:** General status of dairy production in the 15 countries from three regions of sub-Saharan Africa

Category	Average	Standard deviation	Coefficient of Variation (CV %)
Number of exotic breeds	2.8	1.66	59
Number of indigenous breeds	2.2	1.52	69
Milk consumed as liquid (%)	79.0	26.01	33
Milk processed (%)	21.0	12.36	53
Number of dairy production systems	4.3	3.85	89
Number of land tenure/ownership systems	2.1	1.32	63

There were significant differences in the dairy breeds used for dairy production in the regions (*P* < 0.01). The predominant dairy breeds used for dairy production across the regions and countries as identified by respondents were mainly Holstein-Friesian, Jersey, Brown Swiss, Sahiwal, indigenous Zebu breeds (White Fulani, Nguni and Tuli) and their crosses at various levels. However, the number of these breeds used varied in the regions and countries. Either pure- or crossbreeding of foreign and indigenous dairy breeds were practised at different levels in the various production systems. This helped in increasing milk yield in crossbred cattle. In general, there were four main dairy production systems practised in the three regions and across the countries as identified by the respondents. These systems are mixed crop-livestock systems, intensive (zero-grazing) systems, subsistence farming systems and pastoral and pasture-based systems. In the mixed systems, crops were a primary source of food for farmers and their families, and the sale of crops provided income, while residues from the crops were used to feed their cattle. Predominant land tenure systems used across the three regions included: private, communal, group ownership and lease-hold. The number of systems used was significantly different across regions as illustrated in Table 2.

**Table 2:** Number of dairy breeds and dairy systems in different regions of SSA (marginal means from linear model)

	Dairy breeds			Dairy systems				
	Exotic		Indigenous		Production systems		Land tenure systems	
Regions	Frequency	s.e	Frequency	s.e	Frequency	s.e	Frequency	s.e
Eastern	3.2 ^α^	0.3	2.1	0.3	3.5	0.2	2.0^α^	0.13
Southern	4.7 ^β^	0.5	2.1^α^	0.4	3.7	0.3	2.5^β^	0.22
Western	2.2^β^	0.3	3.6^β^	0.3	4.0	0.2	2.2^β^	0.15

Different superscript in each trait denotes significant differences between regions (*P* < 0.05)

^α^Significant

^β^Significant with other regions

### Human capacity in sub-Saharan Africa dairy improvement

**Fig. 1:**
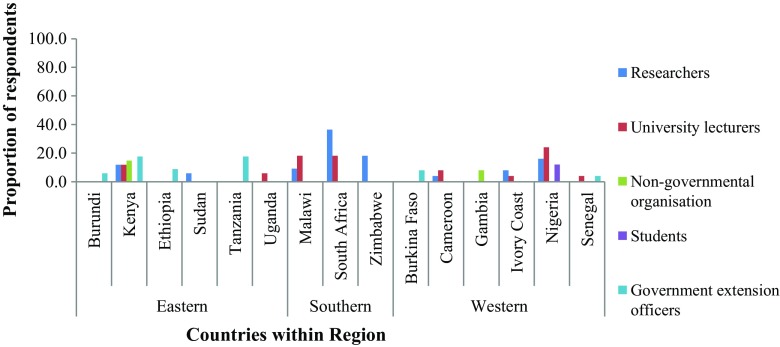
Existing capacity of respondents by country and region

Respondents were self-identified hence researchers were defined as individuals working with mostly research institutes, private companies or as consultants; lecturers were defined as those working in universities and other higher education institutes; government extension workers were defined as those working in extension services such as animal husbandry; non-governmental workers were defined as those working with parastatals not owned/funded by a country’s government; and students were defined as those undergoing training in universities and higher education institutes in the fields of animal science, genetics and veterinary medicine. Researchers, lecturers and government extension officers each accounted for 29% of the respondents, 10% worked with non-governmental parastatal organisations while 3% were students. Figure 1 shows the existing capacity in terms of respondent’s occupation and their involvement in dairy practises in 15 countries by the regions in sub-Saharan Africa.

Across all the regions, Eastern Africa had the highest capacity (48.6%), followed by Western Africa (35.7%) then Southern Africa (15.7%). Among the East African countries, Kenya had the most response rate (27%). In West Africa, Nigeria had the highest response rate (~19%) while in Southern Africa, South Africa had the highest response rate (~9%). Among the participating countries Nigeria, Kenya and South Africa had higher numbers of researchers, lecturers and non-governmental workers.

### Market organisation in sub-Saharan Africa

**Fig. 2:**
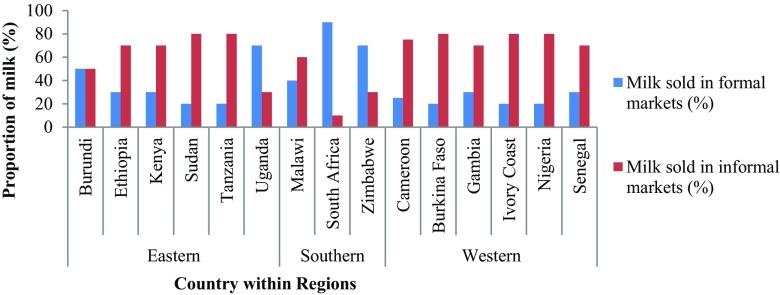
Proportion of milk sold in formal and informal markets in countries of SSA

The structure and organisation of the dairy market varied significantly across the respondent’s countries and geographic regions (*P* < 0.01, Fig. 2). Dairy processors in South Africa handled the highest proportion of milk (24%) and also sold more milk to the formal markets (67%). In Western Africa, most of the milk was sold in the informal market (76%).

The proportions of milk sold in informal markets were higher (60%) than that sold in formal markets (40%) across all countries. Approximately 80% of milk produced within the SSA countries was consumed as liquid and very little was provided to industries for processing into yoghourts, cheese, ghee, butter and powdered milk products.

### Comparison of the current status of dairy production in the different countries and regions of SSA

Marginal averages for existing status of dairy production in one country (Burkina Faso as reference) were compared with the other 14 countries (Table 3). The existing dairy production status varied across the countries (*P* < 0.05). The production systems and land tenure systems in which dairy production was practised tended to be similar and not significantly different between any two countries. However, when countries were grouped into regions, dairy production systems and the status of dairy production were significantly different (*P* < 0.05). The land tenure systems in which dairy production was practised were not significantly different across countries and regions (Table 3). Eight countries (BR, CA, GA, NG, SE, SA, TZ and UG) had significantly higher liquid milk consumption than average while two countries (BF and SU) had significantly lower milk consumption (40% and 20%, respectively) (*P* < 0.05). BF and BR had significantly lower than average proportion of milk sold in formal markets (20%) compared with a higher proportion in ZW (70%) (*P* < 0.05). Table 3 summarises the results.

**Table 3:** Comparison among countries regarding current status of dairy production and marketing routes (Marginal means from linear model analyses)

Region	Country	Milk liquid (%)	Milk processed (%)	Formal market (%)	Informal market (%)	No. of exotic breeds	No. of indigenous breeds	No of production systems^θ^	No. of land tenure systems^θ^
Eastern	BR	95.0 (5.0)^α^	2.5 (5.0)^β^	47.5 (4.0)^α^	52.5 (4.0)^α^	1.0 (0.37)^β^	1.0 (0.4)^β^	4.0 (0.5)	1.0 (0.2)
	ET	85.0 (4.1)^α,β^	15.0 (4.0)^β,θ^	31.7 (3.3)^β^	68.3 (3.3)^β^	2.0 (0.31)^β^	1.0 (0.3)^β,θ^	1.0 (0.4)	2.0 (0.1)
	KE	88.6 (1.6)^α,β^	11.4 (1.6)^β^	32.5 (1.3)^β^	67.5 (1.3)^θ,β^	4.3 (0.12)^α,β^	2.3 (0.1)^α^	3.7 (0.2)	2.0 (0.1)
	SU	20.0 (5.0)^α^	80.0 (5.0)^β^	20.0 (4.0)^β^	20.0 (4.0)^α,β^	1.0 (0.37)^β^	5.0 (0.4)^α^	3.0 (0.5)	1.0 (0.2)
	TZ	88.3 (2.9)^α^	11.7 (2.9)^β^	22.5 (2.3)^β^	2.3 (3.3)^β^	2.3 (0.22)^β^	1.5 (0.2)^β^	4.0 (0.3)	3.0 (0.1)
	UG	100.0 (5.0)^α^	0.0 (5.0)^β^	70.0 (4.0)^α,β^	30.0 (4.0)^α,β^	2.0 (0.37)^β^	1.0 (0.4)^β^	4.0 (0.5)	1.0 (0.2)
Southern	ML	58.3 (4.1)^β^	41.7 (4.0)^α,θ^	30.0 (3.3)^β,θ^	70.0 (3.3)^βθ^	1.0 (0.31)^β^	2.0 (0.3)^β^	4.0 (0.4)	2.0 (0.1)
	ZM	65.0 (5.0)^β^	35.0 (5.0)^θ^	70.0 (4.0)^α^	30.0 (4.0)^α^	4.0 (0.37)^α,β^	5.0 (0.4)^α^	3.0 (0.5)	4.0 (0.2)
	SA	98.0 (2.9)^α^	2.0 (2.9)^β^	82.5 (2.3)^α,β^	17.5 (2.3)^α,β^	6.8 (0.22)^α,β^	1.2 (0.2)^β^	3.8 (0.3)	2.2 (0.1)
Western	BF	40.0 (5.0)^α^	0.0 (5.0)^α,β^	20.0 (4.0)^α^	80.0 (4.0)^α^	2.0 (0.37)^α^	1.0 (0.4)^β^	4.0 (0.5)	1.0 (0.2)
	GA	100.0 (5.0)^α^	0.0 (5.0)^β^	30.0 (4.0)^β^	70.0 (4.0)^θ^	3.0 (0.37)^β^	1.0 (0.4)^α^	1.0 (0.5)	1.0 (0.2)
	IC	58.3 (4.1)^β^	41.7 (4.0)^α^	23.3 (3.3)^β^	76.7 (3.3)^β^	3.0 (0.31)^β,θ^	3.0 (0.3)^α^	5.0 (0.4)	4.0 (0.1)
	NG	94.6 (2.0)^α^	5.4 (1.9)^β^	1.6 (1.6)^β^	74.6 (1.6)^β^	2.0 (0.15)^β^	4.6 (0.2)^α^	3.9 (0.2)	2.0 (0.1)
	SE	100.0 (5.0)^α^	0.0 (5.0)^β^	27.5 (4.0)^β^	72.5 (4.0)^β,θ^	2.5 (0.37)^β^	5.0 (0.4)^α^	5.5 (0.5)	2.5 (0.2)
	CA	100.0 (4.1)^α^	0.0 (4.0)^β^	23.3 (3.3)^β^	76.7 (3.3)^β^	1.3 (0.31)^β^	2.7 (0.3)^β,θ^	4.0 (0.4)	2.0 (0.1)

*BF* Burkina Faso; *BR* Burundi; *CA* Cameroon; *ET* Ethiopia; *GA* Gambia; *IC* Ivory Coast; *KE* Kenya; *ML* Malawi; *NG* Nigeria; *SE* Senegal; *SA* South Africa; *SU* Sudan; *TZ* Tanzania; *UG* Uganda; *ZM* Zimbabwe

^α^Highly significant

^β^Significant with other countries

^θ^Not significantly different from other countries

### Key issues facing dairy production in 15 countries of sub-Saharan Africa

Key issues impacting dairy production in the countries differed significantly across the regions (Eastern, Western and Southern Africa) and 15 countries (*P* < 0.05). Respondents identified the main factors affecting dairy production as (i) poor quality of imported exotic breeds and their crosses in Africa (62.3%), (ii) fluctuations in milk prices in both formal and informal markets (50.9%), (iii) inadequate genetic evaluations of individual animals and sires (39.6%), (iv) poor management systems in terms of herd health, feeding and housing (32.1%) and (v) poor infrastructural facilities (30.3%). Poor animal identification and recording, inadequate breeding policies and sire ranking systems, and lack of systematic performance and pedigree recording were integrated into genetic evaluation factors. Inadequate market road network, poor storage and poor processing facilities among others were incorporated into infrastructural facilities.

## Discussion

There are still several challenges of dairy production in SSA which need to be addressed so as to meet the target for demand and supply of milk by 2025 (Delgado 2005) and 2050 (Alexandratus and Bruinsma 2012). Key issues facing African dairy production as highlighted through the survey have also been reported in the literature (Steinfield et al. 2006; Ehui et al. 2009; Muia et al. 2011). Inadequate uptake of genetic technologies and poor genetic assessment of imported and foreign (exotic) breeds and crosses also hinders dairy improvement in Africa (Nielsen et al. 2013). In most countries of SSA, dairy cows in small-scale farms are exposed to poor feeding (Martin-Collado et al. 2015), housing and health management regimes which adversely affect milk yield (Delgado 2005). There is a great need to improve dairy production and performance of both foreign (exotic), local and crossbred animals. Across-country genetic assessment of exotic, indigenous breeds and their crosses in SSA has been proposed as a means of enhancing dairy production and achieving genetic gains (van Marle-Kӧster and Webb 2014). Challenges in the marketing of milk also hinder progress in dairy productivity. Inadequate access to the milk markets by dairy farmers is generally attributed to bad road networks and poor transport facilities. Milk wastage and spoilage results from poor storage facilities and interruption of the milk supply chain by numerous marketing agents before reaching the consumer (Staal 2006). The prices of milk and milk products and market-support services are not streamlined (Willemse 2011). Milk prices in most countries fluctuate due to unorganised small-scale farm businesses which serve as a key in informal market for milk (Staal 2006).

Solutions to mitigating the challenges in SSA in addition to focusing on aspect around the animals reared should include components related to infrastructural development and enhancing the capacity of different levels of actors in the dairy value chain. Improving the availability of quality feed and feed resources for dairy animals is a critical step in improving milk production. Dairy farmers should also have access to more productive and resilient breeds and breeding animals based on evidence generated through information collated on the animals that are adequately evaluated (Bebe et al. 2003). Systems for recording animal performance and providing feedback to farmers need to be developed with supportive policies to enable their large-scale adoption. Support and involvement of both public and private sector actors in the dairy sector is needed. This could be achieved through partnerships between dairy farmers, service providers and industry stakeholders (Kurwijila 2002). Within the different countries in each region of SSA, the dairy industry will have to organise itself in order to address the challenges with a futuristic outlook. Awareness and enlightenment of existing and prospective dairy farmers in Africa on technologies and innovations that can positively impact dairy production is crucial. This could be facilitated through the establishment of learning hubs and training centres, and the use of cooperating groups as outlined in Ojango et al. (2017).

## Conclusion

This paper highlighted the differences between the current status of dairy production systems, dairy improvement infrastructure, genetic improvement techniques and human capacity in the different regions of sub-Saharan Africa. The challenges, though similar across the countries and regions, differed in magnitude across the regions. It was evident that the quantity of milk available in the different countries greatly influenced the nature of marketing of milk and milk products. Where milk quantities were lower, there was a high dominance of informal milk markets. Sustainable change in dairy productivity in SSA will require information sharing among different actors in the dairy value chain and increased collaboration among the countries in terms of adapting innovations and sharing lessons learnt. As alluded to by respondents from the different regions of the continent, common breeding schemes across countries if collaboratively implemented could revolutionise dairy production in sub-Saharan Africa.

## Implication

Dairy production in sub-Saharan Africa promises a huge potential to improve the farmers’ income and contribute to the overall rural development and prosperity if there is an adequate dairy infrastructure in place. This paper contributes to the knowledge required for food security through the optimisation of across-country breeding schemes for dairy production in sub-Saharan Africa.
